# Fungal Ligninolytic Enzymes and Their Application in Biomass Lignin Pretreatment

**DOI:** 10.3390/jof9070780

**Published:** 2023-07-24

**Authors:** Anna Civzele, Alise Anna Stipniece-Jekimova, Linda Mezule

**Affiliations:** Water Research and Environmental Biotechnology Laboratory, Water Systems and Biotechnology Institute, Faculty of Civil Engineering, Riga Technical University, LV-1048 Riga, Latvia; alise-anna.stipniece-jekimova@rtu.lv (A.A.S.-J.); linda.mezule@rtu.lv (L.M.)

**Keywords:** lignocellulosic biomass, biomass pretreatment, ligninolytic enzymes, white rot fungi

## Abstract

Lignocellulosic biomass is a significant source of sustainable fuel and high-value chemical production. However, due to the complex cross-linked three-dimensional network structure, lignin is highly rigid to degradation. In natural environments, the degradation is performed by wood-rotting fungi. The process is slow, and thus, the use of lignin degradation by fungi has not been regarded as a feasible technology in the industrial lignocellulose treatment. Fungi produce a wide variety of ligninolytic enzymes that can be directly introduced in industrial processing of lignocellulose. Within this study, screening of ligninolytic enzyme production using decolorization of ABTS and Azure B dyes was performed for 10 fungal strains with potentially high enzyme production abilities. In addition to standard screening methods, media containing lignin and hay biomass as carbon sources were used to determine the change in enzyme production depending on the substrate. All selected fungi demonstrated the ability to adapt to a carbon source limitation; however, four strains indicated the ability to secrete ligninolytic enzymes in all experimental conditions—*Irpex lacteus*, *Pleurotus dryinus*, *Bjerkandera adusta*, and *Trametes versicolor*—respectively displayed a 100%, 82.7%, 82.7%, and 55% oxidation of ABTS on lignin-containing media and 100%, 87.9%, 78%, and 70% oxidation of ABTS on hay-containing media after 168 h of incubation. As a result, the most potent strains of fungi were selected to produce lignocellulose-degrading enzymes and to demonstrate their potential application in biological lignocellulose pretreatment.

## 1. Introduction

The rapid growth of the global population over the past half-century has led to an increasing demand not only for fresh water and food but also for petroleum products [1]. At present, energy consumption and production contribute to two-thirds of global emissions, and 81% of the global energy system is still based on fossil fuels, the same percentage as 30 years ago [2,3]. The current aim of the European Union (EU) is to become carbon neutral with net-zero greenhouse gas emissions by 2050 [4]. Renewable biomass will continue to have an important role in EU energy, covering approximately 5% of the primary energy supply of the EU-27 [5]. Generally, lignocellulosic biomass consists of three main components—cellulose (40–50%), hemicellulose (25–30%), and lignin (15–20%), as well as extractives (0–15%), and the proportions of these vary depending on the biomass source [6,7]. From these, lignin is an aromatic biopolymer found in the vascular tissues of plants, and together with cellulose and hemicellulose, forms a natural structural bio-composite, which provides rigidity and mechanical strength to the plant’s cell and structures [8]. The composition of lignocellulosic biomass varies based on the source of the biomass and type of the plant (Table 1); it is also released as a by-product in the pulp, paper, and bioethanol industries. The pulp industry produces around 30 million tons of lignin per year [9], which makes the industry a large producer of lignocellulosic and lignin waste. Lignin, both from plant biomass and industrial waste sources, can serve as an important renewable raw material source in a variety of other commercial applications. Biofuel production, as well as the biological production of chemicals, are potential applications for the biodegradation of lignin.

Most of the applications of lignocellulosic biomass require pretreatment to partly or completely degrade lignin [10]. However, in addition to providing plant stems the rigidity and waterproofing vascular tissues for sap circulation, the role of lignin is the protection of the cellulose polymer towards hydrolytic attack by saprotrophic organisms, which makes the lignin treatment and degradation process difficult. Nevertheless, some microorganisms have developed a strategy to be able to degrade lignin based on unspecific one-electron oxidation of the benzenic rings in the different lignin substructures by extracellular ligninolytic enzymes [11]. Given the role of ligninolytic enzymes in the lignocellulosic biomass degradation and detoxification in the environment [6,12], the biological pretreatment could be a possible environmentally friendly and chemical-free method for the degradation of lignin and lignin biowaste in many industries [6].

**Table 1:** Composition of lignocellulosic biomass from various substrates [13,14,15,16,17,18].

Lignocellulosic Materials	Cellulose (%)	Hemicellulose (%)	Lignin (%)
Natural biomass sources			
Hardwood	40–55	24–40	18–25
Oak	43.2	21.9	35.4
Pine	45.6	24	26.8
Natural hay	44.9	31.4	12
Leaves	15–20	80–85	0
Reed	34–36	26–27	21
Switchgrass	31–45	20–31	12–18
Agricultural biomass by-products or residues			
Barley straw	31–45	27–38	14–19
Wheat straw	33–38	26–32	17–19
Rye straw	33–35	27–30	16–19
Oat straw	31–37	27–38	16–19
Silage	39.27	25.96	9.02
Hemp	53.86	10.6	8.76
Rapeseed	20–35	15–22	15–23
Industrial residues and waste			
Willow sawdust	35.6	21.5	28.7
Paper	85–99	0	0–15
Newspaper	40–55	25–40	18–30
Waste papers from chemical pulps	60–70	10–20	5–10
Primary wastewater solids	8–15	NA	24–29
Sugarcane bagasse	44	28	21

Numerous fungi, as well as bacterial species, can cause lignin degradation (Table 2); however, fungi are more efficient in the breakdown of lignin than bacteria, which are more limited in their enzyme secretion abilities and lignin degradation rates [6]. It has been reported that around 1600–1700 wood-rot fungal species identified in North America are capable of biodegrading lignin [19]. Wood-degrading species are mostly saprotrophs or weak parasites in forest ecosystems. Saprotrophic fungi associated with lignin degradation have been divided into the following three major groups depending on their morphology and enzymes associated with the lignin degradation mechanism: white rot, brown rot, and soft rot fungi. All three groups of fungi are able to degrade lignin, but only Basidiomycota (aerobic white rot fungi) are able to decompose lignin completely to CO_2_ and H_2_O [20,21]. In nature, white rot fungi mostly occur on hardwoods and are primary lignin degraders, whereas brown rot fungi more often are found on softwoods in coniferous ecosystems [22].

The enzymes involved in lignin degradation have been divided into two groups—lignin-modifying enzymes (LME) and lignin-degrading auxiliary (LDA) enzymes [37]. LMEs are also called ligninolytic enzymes and have gained attention as biological agents for the degradation of lignocellulosic waste-containing compounds and other organic pollutants [12]. These enzymes also have a role in industrial waste treatment and other xenobiotic compounds through the biodegradation and decolorization process [37]. The LDA enzymes are unable to degrade lignin on their own and need additional enzyme involvement for complete lignin degradation; however, these enzymes enable the process of lignin degradation through the sequential action of several proteins that may include oxidative H_2_O_2_. This group includes cellobiose dehydrogenase, aryl alcohol oxidases, glyoxal oxidase, glucose oxidase, and pyranose 2-oxydase [12].

LME produced by microorganisms are classified as phenol oxidases (laccases) and heme-containing peroxidases—lignin, manganese, and versatile (multifunctional) peroxidase [38,39]. Recently, a new superfamily of heme peroxidases called dye-peroxidases (DyP, originally named dye-decolorizing peroxidases) was identified in fungi and later in bacteria. DyP may also have a role in the lignin degradation process; however, the clear mechanisms behind their abilities are yet to be discovered [40].

The lignin peroxidases (LiP) are capable of attacking lignin polymers and are relatively non-specific to their substrate. These enzymes are characterized by their ability to oxidase different phenolic aromatic compounds as well as a variety of non-phenolic lignin model compounds and other organic molecules [38]. LiPs were first discovered in the 1980s in *Phanerochaete chrysosporium* and later in *Trametes versicolor*, *Bjerkandera* sp., and *Phlebia tremellosa*, which are well-known white rot fungi species [37]. Microorganism-secreted enzymes are usually a family of isozymes whose relative composition and isoelectric points vary depending on growth conditions, culture media, and nutrients provided in the cultivation process [41].

Similarly to LiP, manganese peroxidases (MnP), as a family of isozymes, were first discovered over 30 years ago and were first detected in *P. chrysosporium*. MnPs are another important LME and have also been found in other Basidiomycota species, including *Panus tigrinus*, *Lenzites betulinus*, *Agaricus bisporus*, *Bjerkandera* sp., and *Nematoloma frowardii*. Details about MnPs presence in bacteria, yeast, and mold are emerging in the scientific literature, and the presence and activity of these enzymes have been studied in several species under the absence and presence of enzyme inducers [37].

Versatile peroxidases (VPs) combine the molecular architecture of LiP and MnP and oxidize typical LiP substrates as well as Mn^2+^, yet they also oxidize azo-dyes and other non-phenolic compounds with high redox potentials in the absence of mediators [42,43]. VPs were first found in members of the genera *Pleurotus* (*P. eryngii*, *P. ostreatus*) and *Bjerkandera* (*B. adusta*, *B. fumosa*) [37].

Dye-decolorizing peroxidases (DyP) are a new family of heme peroxidases, phylogenetically unrelated to other LME peroxidases [44]. These enzymes were first discovered in a culture of the fungus *B. adusta* and, as the name suggests, are able to decolorize a wide range of dyes [40]. The ligninolytic activity of DyP has been reported in other fungi (*Termitomyces albuminosus*, *Auricularia auricula-judae*, and *Irpex lacteus*) and several bacterial species [37]. The presence of DyP-expressing genes is more common in bacteria, and a smaller number of genes are reported in fungi and higher eukaryotes and archaea, suggesting that these enzymes are the bacterial equivalent of fungal LME [45]. DyPs are relatively non-specific to their substrate and oxidase all typical peroxidase substrates as well as have an additional hydrolase or oxygenase activity [46]. DyP are active at lower pH values (pH range 3–4) and are able to degrade different dyes; however, the physiological role of these enzymes is still unclear [47].

Laccases are considered the most important components of the lignin degradation process and are widely distributed in plants, fungi, bacteria, and insects; however, the role of laccases in these processes is not known in detail. All laccases oxidize a range of aromatic compounds, phenolic components also found in lignin, aromatic amines, benzenothiols, and hydroxyindols using molecular oxygen as an electron acceptor, bypassing a stage of hydrogen peroxide production [37,48]. These enzymes are extracellular, periplasmic, and intracellular proteins, and a majority of fungi produce mostly extracellular as well as some intracellular laccases [48]. In plants, intracellular laccases participate in the synthesis of lignin, intracellular fungal laccases, and periplasmic bacterial laccases most likely participate in the transformation of phenolic compounds in the cell, while extracellular laccases participate in lignin degradation [49,50]. Fungal laccases also participate in the pathogenesis, detoxification, and development of higher fungi [51]. Laccases belong to the group of polyphenol oxidases and are also called blue multicopper oxidases due to containing copper atoms in the catalytic site of the enzyme [51], and mainly react with free phenolic fragments of lignin due to the random polymer nature of lignin and laccases lower redox potential; however, mediators can cause a reaction to non-phenolic compounds with higher redox potential [52]. Similarly, as peroxidases, laccases are also secreted as several isoforms in most fungi, originating from the same or different genes. The number and properties of isozymes secreted vary depending on the growth conditions, fungal species, as well as nutrients and inducers found in the growth media [37,48].

In recent years, ligninolytic enzymes have gained applications in the fields of the food industry, textile industry, synthetic chemistry, cosmetics, soil bioremediation and biodegradation of environmental phenolic pollutants, and removal of endocrine disruptors [53,54]. These enzymes are also used for paper and pulp delignification, where they can be used in the enzymatic adhesion of fibers in the manufacturing of lignocellulose-based composite materials, such as fiberboards [54]. Using fungal strains, which can produce the enzymes needed for biomass conversion and to produce ethanol, improved biorefinery efficiency can also be achieved [55,56].

To evaluate the ability of fungal growth and lignocellulosic biomass degradation, mostly culture media or pure lignin in culture media has been used [40,57,58,59]. Here we report a screening study of 10 biotechnologically relevant fungal isolates on their ability to produce lignin-degrading enzymes in the presence of untreated lignocellulosic biomass.

## 2. Materials and Methods

### 2.1. Microorganisms

In this study, commercially available cultures of *Irpex lacteus* DSM 9595, *Pleurotus dryinus* (Pers.) *P. Kumm*, *Pleurotus ostreatus* DSM 1020, *Bjerkandera adusta* DSM 23426, *Trametes versicolor* DSM 6401, *Pycnoporus cinnabarinus* (Fr.) *P. Karst*, *Aspergillus brasiliensis* ATCC^®^16404™, and cultures isolated from pine forests of Latvia—*Fusarium graminearum*, *Fomitopsis pinicola*, *Trichoderma paraviridescens*, which were maintained on potato dextrose agar (PDA) (Oxoid Ltd., Basingstoke, Hants, UK) medium at 2–8 °C, were used in ligninolytic enzyme screening tests.

### 2.2. Media Conditions and Screening of Ligninolytic Enzymes

To detect the ability of selected fungal species for ligninolytic enzyme production, 0.1% (*w*/*v*) ABTS (2,2′-Azino-bis(3-ethylbonzotiazoline-6-sulfonic acid)) diammonium salt) [60], and 0.01% (*w*/*v*) Azure B [61] were used as reaction substrates. ABTS is a non-phenolic dye that is oxidized by laccase to the more stable and preferred state of ABTS cation radical. The radical is responsible for the distinct blue-green color and can be correlated to laccase activity [62]. A distinct purple color formation has been described when the laccase content was equal to or higher than needed for the reduction of ABTS present [63]. Azure B is a triarylmethane dye and has a similar structure to lignin, thus is usually used as the substrate to measure the ligninolytic enzyme activity. Decolorization of this dye illustrates the presence of LiP, MnP, or laccase produced by the fungi [64].

To prepare agar medium containing lignin or hay, 2 g of the respective substrate, 0.8 g KH_2_PO_4_, 0.4 g K_2_HPO_4_, 0.5 g MgSO_4_·7H_2_O, 2 g NH_4_NO_3_, 2 g yeast extract, and 15 g agar (pH 5.5 ± 0.2) were added per L of distilled water. The prepared agar media were sterilized by autoclaving at 121 °C for 15 min. The chemical composition of the hay biomass from grasslands, which includes approximately 22–26% cellulose, 14–25% hemicellulose, and 1–13% lignin, has been adopted for this study [65].

After media preparation, 1 cm^2^ mycelial disk of each fungal species was placed on 9 types of agars (Table 3) containing PDA, lignin (Sigma-Aldrich, Darmstadt, Germany) or hay (dry weight (DW): 92.8 ± 1.3%, collected from semi-natural grassland in Latvia) as biomass substrate. The specific initial color of the media was recorded (Table 3).

During the screening tests, agar plates were incubated for 168 h at 25 °C and 80% rH in constant climate chamber (KBF 115, BINDER GmbH, Tuttlingen, Germany). Oxidation zone and dark color formation around the mycelium indicated on the presence of ligninolytic enzymes. The diameter and intensity of the color change were used as an indicator of the lignocellulosic enzyme production. Color zone formation and color change intensity were monitored and captured daily using NIKON D3300 (NIKON, Tokyo, Japan). Visual analysis was performed to determine the qualitative changes in the agar plates. Quantitative analysis was conducted to determine the percentage of the agar plate area that was covered by fungal mycelium and underwent oxidation induced in the presence of fungal enzymes by measuring the diameter of the fungal mycelium and the oxidation zone. The experiments were performed in 3 independent repetitions.

## 3. Results and Discussion

**Figure 1:**
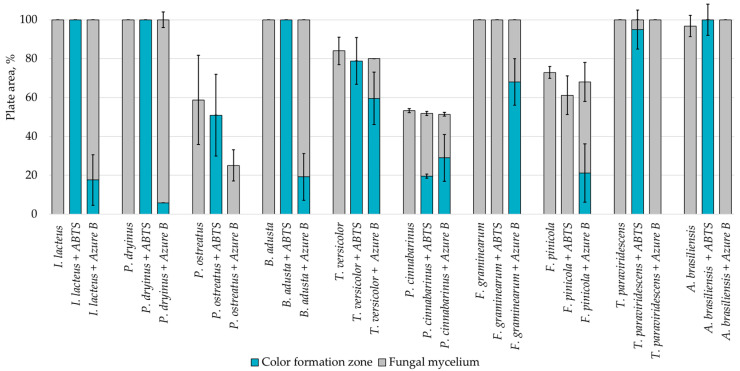
Percentage of grown fungal mycelium and formed oxidation zone of the total PDA agar area after 168 h of incubation.

Fungal growth and substrate oxidation results show that on PDA, within 168 h, four fungal strains—*I. lacteus*, *P. dryinus*, *B. adusta,* and *T. paraviridescens*—fully covered the agar plate and simultaneously showed the most intense ABTS oxidation (Figure 1). However, only one white rot fungus—*T. versicolor*—caused significant color change on both ABTS (78.8% of plate area) and Azure B (59.6% of plate area) agar.

**Figure 2:**
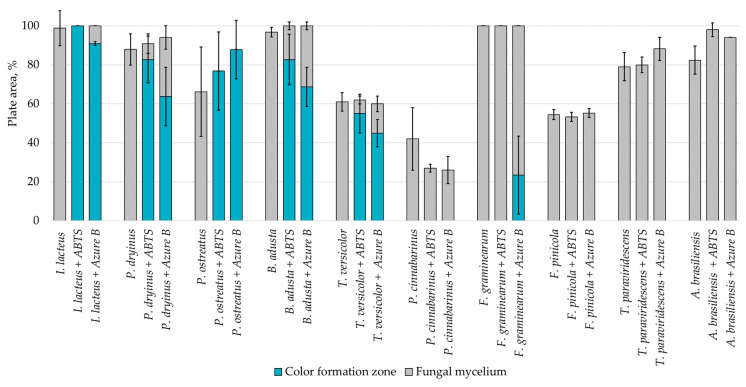
Percentage of grown fungal mycelium and formed oxidation zone of the total lignin agar area after 168 h of incubation.

Agar containing only lignin as a carbon source limited the fungal growth rate, and only *F. graminearum* was able to fill the agar plates completely (Figure 2) in both PDA and lignin-containing medium. The mycelium growth rate of *I. lacteus* and *B. adusta* was less influenced by a change in carbon source. The growth activity of *I. lacteus* was decreased only by 1.2% and *B. adusta*—only by 3.2%. A more significant decrease in mycelium growth was observed in *T. paraviridescens* (21.0%), *P. cinnabarinus* (21.2%), *F. pinicola* (25.2%), *T. versicolor* (27.4%), and *A. brasiliensis* (48%) cultures. This suggests that different fungal species have varying adaptability and metabolic strategies in response to carbon source limitations.

Despite the limitations imposed by the carbon source, significant color change and ABTS oxidation were still observed in five white rot fungal cultures—*I. lacteus*, *P. drynus*, *P. osteatus*, *B. adusta,* and *T. versicolor*. In contrast to the PDA plates, decolorization of Azure B was found in different intensities in the previously mentioned cultures depending on the media composition, which suggests that carbon source limitation to agar only intensifies enzyme secretion in fungal cultures leading to more efficient decolorization and lignin degradation.

**Figure 3:**
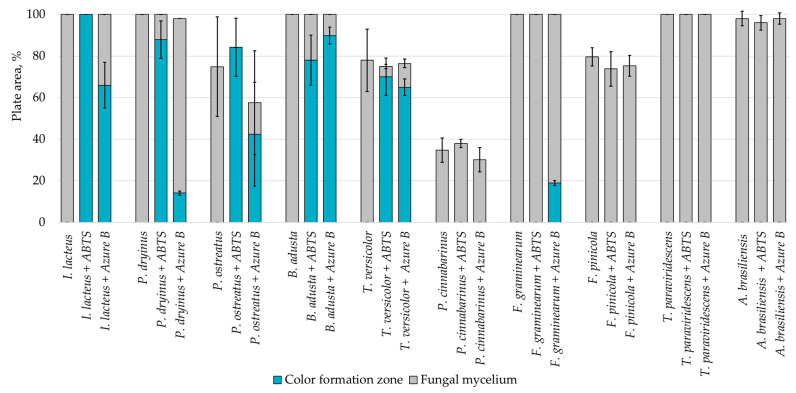
Percentage of grown fungal mycelium and formed oxidation zone of the total hay agar area after 168 h of incubation.

Hay biomass provides a more easily available carbon source for fungal growth when compared to lignin, and numerous cultures fully covered the plate surface within 168 h of incubation (Figure 3). The mycelium growth rate of all white rot fungi was increased—by 1.2% in the *I. lacteus* culture, 3.2% in *B. adusta*, 11.6% in *P. ostreatus*, 12.1% in *P. dryinus,* and up to 21.8% in the *T. versicolor* culture. The most significant increase in the growth activity was observed in *F. pinicola* (31.5%). *I. lacteus*, *P. dryinus*, *B. adusta*, *F. graminearum,* and *T. paraviridescens* were able to fully grow in the given time frame, but *F. graminearum* and *T. paraviridescens* failed to demonstrate significant color zone formation with ABTS or Azure B. Significant color formation zones and decolorization of Azure B were observed with *I. lacteus*, *P. dryinus*, *B. adusta,* and *T. versicolor*. *P. ostreatus* was also able to ensure intense oxidation of both ABTS and Azure B, although, the growth rate of *P. ostreatus* was limited and very variable between experiment repetitions. Previous studies describe *P. ostreatus* as a well-known fungal laccase producer with a high catalytic potential and one of the first cultures to produce a significant amount of DyP enzymes. However, it has been tested only in lignin systems or fungal gene expressions in other organisms to produce these enzymes [40,48]. Therefore, the results gathered in this study suggest that for lignocellulose biomass degradation, other fungal cultures seem more promising, given the screening tests with the hay biomass substrate offer a model closer to possible industrial applications for lignocellulosic biomass treatment.

The screening results on lignin and hay biomass agar plates suggest that cultures of *I. lacteus*, *P. dryinus*, *B. adusta,* and *T. versicolor* have the potential to develop technologies for lignin or lignocellulose biomass degradation.

**Figure 4:**
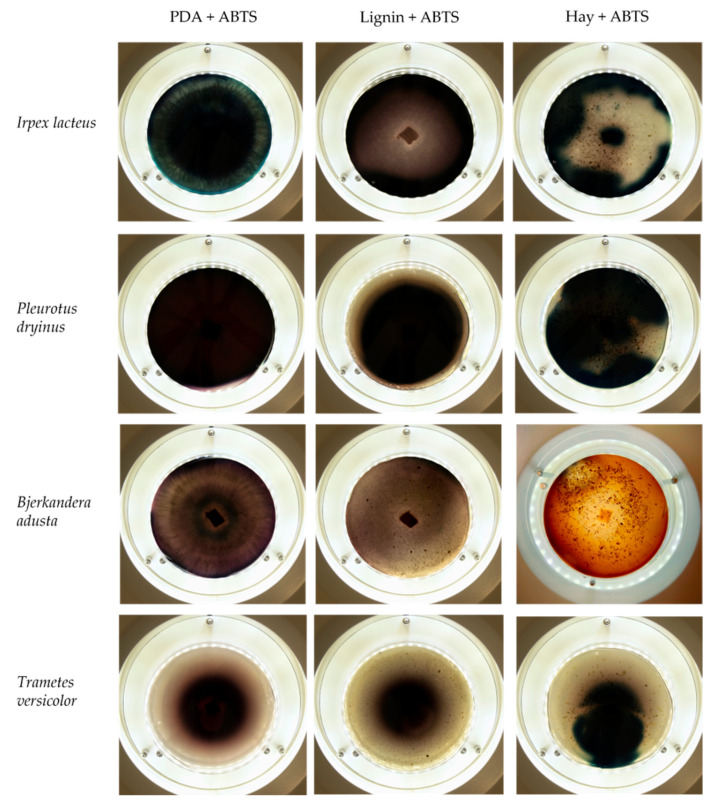
Color formation on ABTS-containing agar by selected fungal strains after 168 h of incubation.

**Figure 5:**
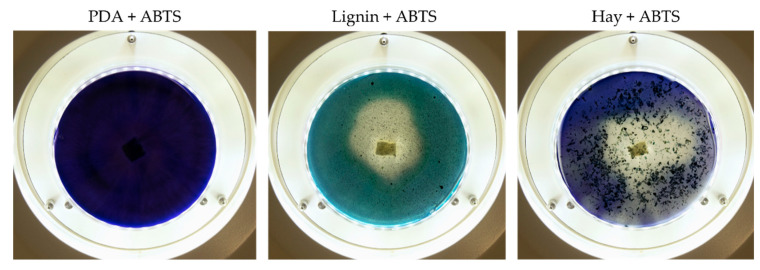
The decolorization of Azure B by *B. adusta* after 168 h of incubation.

Based on the growth rate, color formation zone, and the oxidation intensity of the selected fungi during screening tests, *I. lacteus*, *P. dryinus*, *B. adusta,* and *T. versicolor* were selected as potent fungal species for lignin and lignocellulose biomass treatment. All the selected strains also showed intense color formation during the screening tests using ABTS (Figure 4). However, the most significant color formation on all types of agars was performed by *P. dryinus* and *T. versicolor*. Although, compared to *I. lacteus* and *B. adusta*, the mentioned fungi were characterized by a lower growth rate of the fungal mycelium. The most significant Azure B dye decolorization results were observed using *B. adusta*, the only fungal strain that degraded the added dye completely in the oxidation zone (Figure 5). 

**Figure 6:**
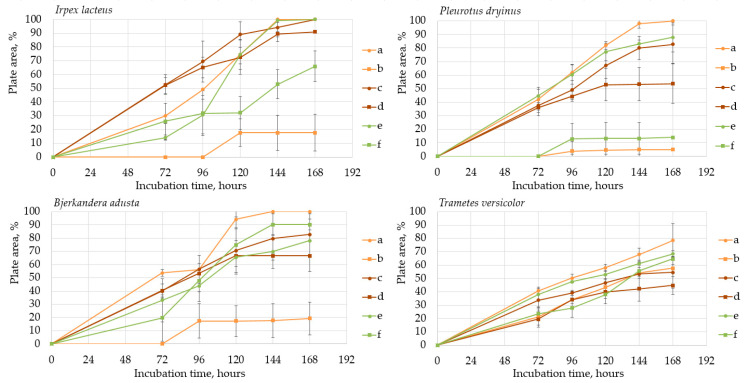
Oxidation zone formation on (a) PDA + 0.1% (*w*/*v*) ABTS; (b) PDA + 0.01% (*w*/*v*) Azure B; (c) lignin agar + 0.1% (*w*/*v*) ABTS; (d) lignin + 0.01% (*w*/*v*) Azure B; (e) hay agar + 0.1% (*w*/*v*) ABTS; (f) lignin + 0.01% (*w*/*v*) Azure B.

When comparing the degradation process of ABTS and Azure B (Figure 6), all four selected fungi caused a faster color formation on the ABTS-containing agar, regardless of the added carbon source. The potential explanation is that the ABTS used in this study for the screening tests has also been studied and used as a mediator that facilitates and promotes the release of lignin-degrading enzymes [59]. The highest rate of color formation occurred on PDA and hay-containing agar. Hay biomass and PDA contain more easily obtainable carbon compounds necessary for fungal growth, which facilitate more rapid fungal growth and enzyme release.

Comparing the ABTS oxidation rate and color formation zone diameter, the fastest color formation was performed by *I. lacteus*, which filled 100% of the plate area within 144 h. The color formation and oxidation of ABTS by *B. adusta* occurred more slowly; however, this culture degraded the color most intensively and efficiently on agar-containing Azure B. This is explained by the fact that *B. adusta* was discovered as a dye-decolorizing peroxidase-secreting fungus and is able to decolorize a wide range of dyes [40]. *T. versicolor* was characterized by the slowest growth compared to *I. lacteus*, *B. adusta,* and *P. dryinus*; however, it showed a high efficiency of the oxidation of both ABTS and Azure B and linear growth and oxidation zone formation rate on all types of agars. *P. dryinus* was characterized by a relatively rapid growth and oxidation zone formation rate on all types of agars, except glucose- and hay-containing medium with Azure B, where the oxidation zone did not exceed 15% of the plate area during 168 h of screening.

## 4. Conclusions

*I. lacteus*, *P. dryinus*, *B. adusta*, and *T. versicolor* demonstrated the most promising results in terms of ABTS and Azure oxidation. These ligninolytic enzyme-producing white rot fungi were identified as potent fungal to offer an environmentally friendly, sustainable, and cost-efficient technology for lignin pretreatment, which could improve lignocellulosic biomass degradation and attain in production of high-value products. No change in the mycelium growth rate of *I. lacteus*, *P. dryinus*, or *B. adusta* on the lignocellulose biomass media was observed, and a decrease of 2.4–8.2% was observed on lignin-containing media when compared to PDA. *T. versicolor* demonstrated a more significant decrease in growth rate; however, all four selected fungal strains formed intense ABTS oxidation zones. Moreover, *B. adusta* displayed efficient decolorization of Azure B dye.

Given the screening results with lignin and lignocellulose substrates, the selected fungal strains can be characterized by the intense release of ligninolytic enzymes and dye oxidation abilities. These findings suggest that these fungal cultures have the potential for developing technologies aimed at lignin or lignocellulose biomass degradation. Further investigation is needed to explore their enzymatic capabilities and optimize their performance for industrial applications in lignocellulosic biomass treatment.

## Figures and Tables

**Table 2:** Bacteria and fungi degradation of lignin on different feedstocks.

Species	Feedstock Used	Lignin Degradation (%)	Time (Days)	Reference
Bacteria				
*Pseudomonas* spp.	Kraft lignin	39	52	[23]
	Poplar wood	40–52	30	[24]
	Kraft lignin	20	40–60	[23]
*Acinetobacter* spp.	Poplar wood	47–57	30	[24,25]
*Xanthomonas* spp.	Poplar wood	39–48	30	[24,25]
*Streptomyces badius*	Indulin lignin	3–4	35	[26]
*Streptomyces viridosporous*	Indulin lignin	3–4	35	[26]
*Streptomyces cyaneus*	Barley straw	29–52	21	[27]
*Thermomonospora mesophila*	Barley straw	36–48	21	[24,27]
Fungi				
*Pleurotus ostreatus*	Cotton stalks	40	30	[28]
	Beech wood	56.5	120	[28]
*Phanerochaete chrysosporium*	Cotton stalks	60	30	[28]
	Cotton stalks	28	14	[23]
*Trametes versicolor* spp.	Beech wood	57.4	120	[29]
	Bamboo culms	9–24	28	[30,31]
*Irpex lacteus*	Cornstalks	15	11.84	[32]
*Echinodontium taxodii* 2538	Bamboo culms	24	28	[30]
*Phlebia* sp. MG-60	Oak wood	40.6	56	[33]
*Ceriporia lacerata*	Red pine	13	56	[34]
*Stereum hirsutum*	Red pine	15	56	[31]
*Ceriporiopsis subvermispora*	Corn stover	39.2	42	[35,36]

**Table 3:** Color of uninoculated agar used in ligninolytic enzyme screening tests.

Substrate	-	0.1% *w*/*v* ABTS	0.01% *w*/*v* Azure B
Potato dextrose agar	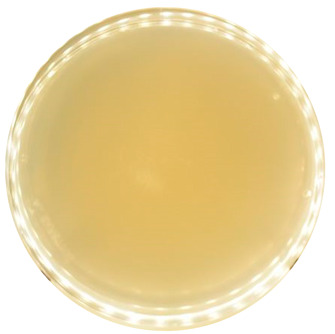	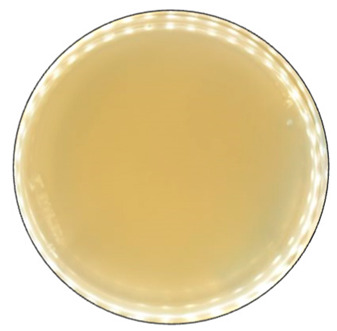	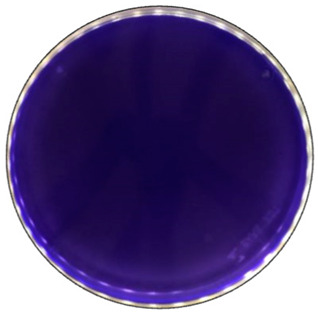
Lignin	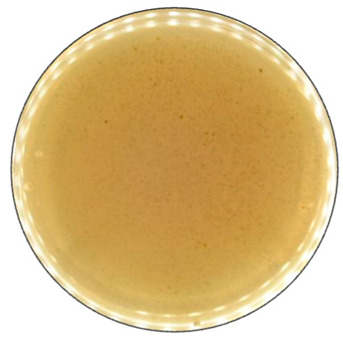	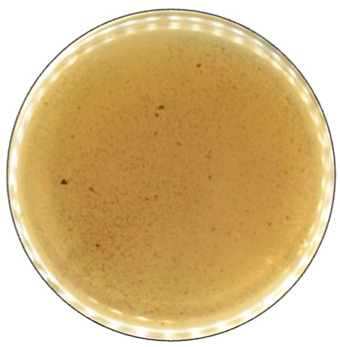	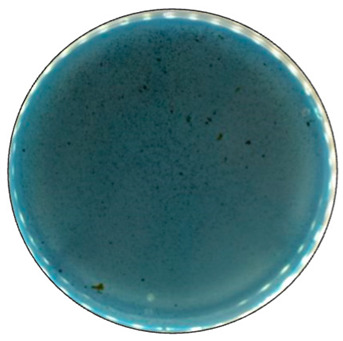
Hay	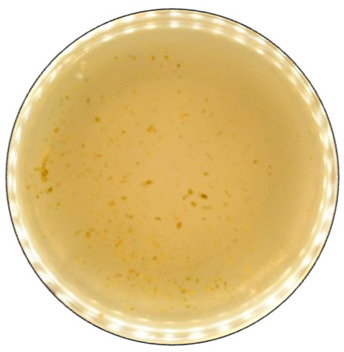	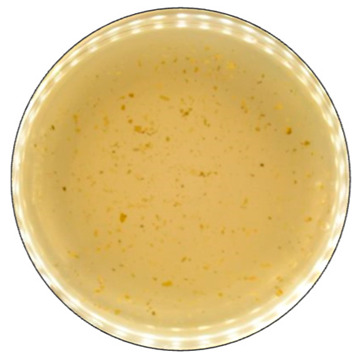	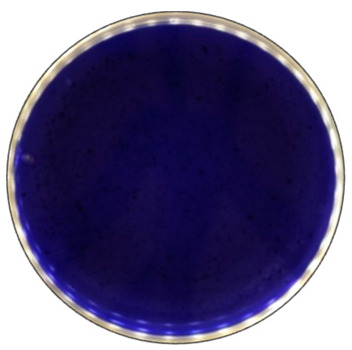

## Data Availability

Not applicable.
